# Fertility Preservation During the COVID-19 Pandemic: Modified But Uncompromised

**DOI:** 10.1089/whr.2021.0107

**Published:** 2022-01-31

**Authors:** Emma Trawick, Elnur Babayev, Nivedita Potapragada, Jennifer Elvikis, Kristin Smith, Kara N. Goldman

**Affiliations:** Division of Reproductive Endocrinology and Infertility, Department of Obstetrics and Gynecology, Northwestern University Feinberg School of Medicine, Chicago, Illinois, USA.

**Keywords:** cancer, controlled ovarian hyperstimulation, COVID-19, fertility preservation, reproductive health

## Abstract

***Purpose:*** Throughout COVID-19, our clinic remained operational for patients requiring urgent fertility preservation (FP). This study aimed to characterize changes to clinical protocols during the first wave of COVID-19 and compare outcomes to historical controls.

***Methods:*** We performed a retrospective cohort study at a university fertility center examining all patients who underwent medically indicated FP cycles during the American Society for Reproductive Medicine (ASRM) COVID-19 Task Force-recommended suspension of fertility treatment (March 17–May 11, 2020) and patients from the same time period in 2019. FP care was modified for safety during the first wave of COVID-19 with fewer monitoring visits and infection control measures. FP cycle characteristics and outcomes were compared across years.

***Results:*** The volume of cycles was nearly 30% higher in 2020 versus 2019 (27 vs. 19). Diagnoses, age, and anti-Mullerian hormone were similar between cohorts. More patients elected to pursue embryo cryopreservation over oocyte cryopreservation in 2020 versus 2019 (45.8% vs. 5.2%, *p* < 0.005). Patients managed during COVID-19 had fewer monitoring visits (5 ± 1 vs. 6 ± 1, *p* = 0.02), and 37.5% of cycles utilized a blind trigger injection. There was no difference in total days of ovarian stimulation (11 ± 1 vs. 11 ± 2, *p* > 0.05), but 2020 cycles utilized more gonadotropin (4770 ± 1480 vs. 3846 ± 1438, *p* = 0.04). There was no difference in total oocytes retrieved (19 ± 14 vs. 22 ± 12, *p* > 0.05) or mature oocytes vitrified (15 ± 12 vs. 17 ± 9, *p* > 0.05) per cycle.

***Conclusions:*** FP continued during COVID-19, and more cycles were completed in 2020 versus 2019. Despite minimized monitoring, outcomes were optimal and equivalent to historical controls, suggesting FP care can be adapted without compromising outcomes.

## Introduction

Fertility preservation (FP) is a critical part of comprehensive cancer care. More than 90,000 women under the age of 45 are diagnosed with cancer each year, and therapeutic advances have led to survival rates >85% with an increased focus on long-term quality of life.^1,2^ As these life-saving treatments often impact fertility, FP can represent patients' only opportunity for autologous reproduction after definitive cancer treatment.^3^ The American Society for Reproductive Medicine (ASRM) and the American Society of Clinical Oncology (ASCO) advise health care providers to review FP with all eligible reproductive-aged patients to ensure patients have the opportunity to pursue FP before definitive gonadotoxic therapy.^4,5^

Standard medical care was sharply disrupted in March 2020 by the onset of the COVID-19 pandemic. The SARS-CoV-2 virus spread rapidly worldwide in just 3 months in early 2020, overwhelming hospital systems across the world and leading to worldwide shutdowns to prevent viral spread. By March 11, 2020, the World Health Organization had declared COVID-19 a pandemic and suggested national governments activate emergency mechanisms to control the virus and divert resources and personal protective equipment (PPE) to frontline health care workers.^6^ While this led to necessary reallocation of resources in the health care systems as COVID-19 spread, it also created tremendous delays for basic medical care.

Given the exponential spread of the virus and its unknown impact on reproduction and pregnancy, as well as the need to divert resources and PPE, on March 17, 2020, the ASRM recommended suspension of new assisted reproductive technology treatment cycles, including intrauterine insemination, *in vitro* fertilization (IVF), and embryo transfer.^7^ Importantly, the ASRM specified that urgent fertility treatment for patients can and should continue, allowing patients facing gonadotoxic chemotherapy or other fertility-threatening medical interventions the opportunity—as well as potential associated risks—to continue care despite the pandemic.

Because time between cancer diagnosis and therapy is often limited, completion of FP requires prompt referral and initiation of treatment. The standard of care for FP, cryopreservation of oocytes and embryos, requires an average of 10–12 days of injectable gonadotropins, 6–7 in-office monitoring visits, and an oocyte retrieval procedure under sedation. COVID-related closures had the potential to create delays in care that would close patients' already-narrow windows to pursue FP.

Our institution continued FP for those with fertility-compromising conditions such as cancer, but as cancer patients are at higher risk of serious morbidity or mortality from COVID-19,^8^ our FP approach was modified for safety by minimizing in-office visits and monitoring. We sought to characterize FP care during the initial COVID outbreak and compare outcomes to historical controls.

## Methods

We performed a retrospective cohort study of all medically indicated FP cycles completed during ASRM's recommended treatment suspension period (March 17–May 11, 2020) compared with cycles from the same time frame in 2019. All included patients had recently been diagnosed with cancer or had another medical condition requiring anticipated gonadotoxic treatment. This study received approval from the Institutional Review Board at Northwestern University (STU00212596).

### Stimulation protocols

Controlled ovarian hyperstimulation (COH) was performed using a gonadotropin-releasing hormone (GnRH) antagonist protocol. The initial dose of gonadotropins (recombinant follicle-stimulating hormone [FSH] and human menopausal gonadotropin; Follistim, Merck; Gonal-F, EMD-Serono; and/or Menopur, Ferring) were prescribed based on each patient's age, antral follicle count, and anti-Mullerian hormone (AMH) levels.

Hormone levels (estradiol [E2], FSH, luteinizing hormone [LH], and progesterone [P4]) and transvaginal ultrasound were used to determine menstrual cycle status for each patient at the time of cycle start, and patients initiated care regardless of menstrual cycle timing. Gonadotropin dose was adjusted based on serum E2 levels and follicle size. Our clinic's standard protocol includes hormone levels and transvaginal ultrasounds beginning 3 days after gonadotropin initiation, with follow-up visits every 2 days or more frequently as clinically indicated. GnRH antagonist (Ganirelix acetate, Organon; or Cetrotide, EMD-Serono) was administered when the lead follicle was ≥13 mm and/or E2 was >300 pg/mL.

Final oocyte maturation was triggered using human chorionic gonadotropin (hCG) and/or GnRH agonist (leuprolide), with planned oocyte retrieval 36 hours after trigger. Oocyte retrieval was performed through transvaginal ultrasound-guided aspiration. Mature (metaphase II, MII) and immature (germinal vesicle) oocytes were cryopreserved on the day of retrieval. MI oocytes were kept in culture overnight and were vitrified the following day if they reached MII stage.

If embryo cryopreservation was planned, intracytoplasmic sperm injection or conventional insemination was performed. Embryos were then either cryopreserved at the two pronuclei (2PN) stage or were cultured to blastocyst stage and then cryopreserved. In our practice, cryopreservation at the 2PN stage is offered to preserve the option of day 3 embryo transfer in the future, if there is low overall yield with the goal to maximize the number of cryopreserved embryos, or if there are plans for future preimplantation genetic testing for monogenic diseases (PGT-M). Otherwise, cryopreservation at the blastocyst stage is preferred. If preimplantation genetic testing for aneuploidy (PGT-A) was desired, trophectoderm biopsy was performed before vitrification on day 5 or 6.

### COVID-19 modifications

During the 2020 COVID-19 pandemic surge described in this study, office protocols were altered to limit possible COVID exposure to patients and staff and optimize the use of available resources. As many consults were scheduled through telehealth as possible, and appointments were consolidated where possible (for example, starting a cycle on the same day as the initial FP visit and baseline monitoring visit). Cycles were initiated with the availability of anesthesia staff in mind, given that anesthesia colleagues were required for essential services, including intensive care units and COVID-19 intubation teams.

Office-wide social distancing measures included significant modifications to the waiting room, including distance between chairs, Plexiglass dividers between reception staff and patients, and markers indicating 6 ft of distance between patients approaching the reception desk. Morning monitoring was performed by appointment only, with no visitors allowed (except in the case of a minor patient).

Sanitation protocols were used to ensure all rooms and surfaces were cleansed thoroughly between patients. Universal masking was employed, and all staff wore N95 masks (when available) in patient-facing areas and N95 masks and eye protection during oocyte retrieval procedures. Staff physically present in the office did not exceed one phlebotomist, one nurse, one sonographer, one physician, anesthesia and nursing staff present on retrieval days as needed, and embryology staff present only as required for embryology procedures.

Clinical decisions were made with the intention of decreasing patients' possible exposures to COVID-19, and these decisions included blindly initiating the GnRH antagonist, making treatment decisions with fewer monitoring visits, and blindly initiating oocyte maturation trigger.

If a patient did not meet clinical criteria for GnRH antagonist initiation, but ultrasound confirmed follicle sizes 11–12 mm with appropriate estradiol levels, GnRH antagonists were initiated in the next 1–2 days without additional confirmatory ultrasound. Patients were then instructed to return when decisions would be necessary regarding trigger timing. For patients whose follicle sizes were not yet appropriate for oocyte maturation trigger, but were anticipated to be appropriate in the subsequent 1–2 days, decisions were made to trigger oocyte maturation without additional confirmatory ultrasound or bloodwork.

Patient at high risk of ovarian hyperstimulation syndrome (OHSS) were monitored with greater frequency as indicated. Clinical judgment was used to ensure sufficient monitoring to achieve optimal patient outcomes while decreasing possible exposures to COVID-19. All decisions regarding oocyte maturation trigger were made in conjunction with anesthesiology colleagues to ensure anesthesiology availability for retrieval given COVID-19 emergency coverage responsibilities. SARS-CoV-2 NAT testing was performed for patients initiating cycles after April 9, 2020, with tests performed no >72 hours before retrieval.

### Outcomes and statistical analysis

Outcomes from included cycles in 2020 were compared with historical controls from the same time period in 2019. We report demographic and clinical characteristics, including the indications for FP. Outcomes measured included the total number of visits, gonadotropin dose used, and the number of oocytes retrieved.

Continuous variables were assessed for normality using D'Agostino–Pearson test; parametric values were compared using student's *t*-test and nonparametric variables were compared using Mann–Whitney *U* test. Categorical variables were compared using Fisher's exact test. The results were adjusted for age, body mass index (BMI), AMH level, and antral follicle count with multiple linear regression. A *p*-value <0.05 was considered statistically significant. Results are reported as mean ± standard deviation, median (range), or percent (%) where appropriate. GraphPad Prism (version 8.0.0 for Mac; GraphPad Software, San Diego, CA) was used for statistical analysis.

## Results

During the first wave of the COVID-19 pandemic in 2020, our center initiated 27 urgent FP cycles for 24 patients. As recommended by ASRM, all other nonurgent fertility treatment cycles were suspended. During this time period, three cycles were cancelled for acutely decompensating lymphoma, no response to gonadotropins in a patient with severely diminished ovarian reserve following prior chemotherapy, and symptomatic COVID-19, respectively. These cycles were excluded from analysis. During the same time period in 2019, 19 cycles were initiated for 19 patients, with no cycles cancelled. External referrals accounted for eight cycles in 2020 (33.3%) and four cycles in 2019 (21.1%, *p* > 0.05).

Baseline characteristics of the patients in 2020 and 2019 are presented in Table 1. Four (4/21 [19.0%]) patients in 2020 had their first visit through telehealth, and no patients in 2019 were seen by telehealth (*p* > 0.05). Insurance plans were similar across cohorts. In 2020, 3/24 (12.5%) cycles were publicly insured by Medicaid compared with 3/19 (15.8%) cycles in 2019 (*p* > 0.05). There were no differences between age and baseline AMH levels between groups. A similar number of patients had partners in both 2020 and 2019 (13/21 [61.9%] vs. 8/19 [42.1%], *p* = 0.34).

**Table 1.** Patient Characteristics in 2020 Versus 2019

	2020 (*n* = 24 cycles, 21 patients)	2019 (*n* = 19 cycles, 19 patients)	*p*
Age	30 ± 7	28 ± 7	0.3
AMH	2.9 ± 2.0	4.2 ± 3.1	0.19
Body mass index	30.3 ± 8.1	27.5 ± 6.8	0.24
Diagnosis			0.73
Breast cancer	7 (29.1%)	7 (36.8%)	—
Leukemia	6 (25.0%)	7 (36.8%)	—
Lymphoma	4 (16.7%)	2 (10.5%)	—
Brain tumor	3 (12.5%)	3 (15.8%)	—
Lupus	2 (8.3%)	1 (5.3%)	—
Gender dysphoria	1 (4.2%)	1 (5.3%)	—
Other malignancy	0 (0%)	2 (10.5%)	—
Other condition	1 (4.2%)	2 (10.5%)	—

Values are expressed as mean ± standard deviation and percentages are represented in parentheses where appropriate.

AMH, anti-Mullerian hormone.

Medical diagnoses and indications for urgent FP in 2020 and 2019 were similarly divided between breast cancer (7/24 [29.2%] vs. 7/19 [36.8%]), leukemia and lymphoma (7/24 [29.2%] vs. 5/19 [26.3%]), and other diseases (10/24 [41.6%] vs. 7/19 [36.8%]) (*p* > 0.05). Six of the patients with breast cancer in 2020 (6/7 [85.7%]) and seven (7/7 [100%]) of the patients with breast cancer in 2019 had estrogen receptor-positive cancers. Of these, two in 2020 (2/7 [28.5%]) and one in 2019 (1/7 [14.2%]) received letrozole during stimulation cycles as per prior clinic protocol.

Fourteen (66.7%) cycles were performed despite unknown COVID status before initiation of routine SARS-CoV-2 NAT testing in April 2020, and the remaining eight (33.3%) cycles were COVID-19 negative before the cycle initiation. One patient's first cycle was cancelled due to symptomatic COVID-19 after routine testing was implemented, but the patient was able to complete a subsequent cycle after recovery with a negative SARS-CoV-2 NAT test. No patients contracted SARS-CoV-2 during FP treatment.

Cycle characteristics and outcomes are shown in Table 2. More embryo freezing cycles were initiated in 2020 than in 2019 (11/24 [45.8%] vs. 1/19 [5.2%], *p* < 0.005). All cycles underwent random start in 2020, and 16 (84.2%) underwent random start in 2019 (*p* = 0.08). Patients undergoing COH during the first wave of COVID-19 in 2020 had significantly fewer total monitoring visits (5 ± 1 vs. 6 ± 1, *p* = 0.02), but with similar total number of days of ovarian stimulation (11 ± 1 vs. 11 ± 2, *p* > 0.05). The total dose of gonadotropins was higher in cycles from 2020 versus 2019 (4770 ± 1480 IU vs. 3846 ± 1438 IU, *p* = 0.04), but the maximum serum E2 levels were similar across cohorts (1667 + 935 pg/mL vs. 1981 + 1089 pg/mL, *p* > 0.05), and antagonist use was similar (6 + 1 days vs. 5 + 1 days, *p* > 0.05).

**Table 2.** Comparison of Controlled Ovarian Hyperstimulation Cycle Outcomes in 2020 Versus 2019

	2020 (*n* = 24 cycles, 21 patients)	2019 (*n* = 19 cycles, 19 patients)	*p*
No. of office monitoring visits^*^	5 ± 1	6 ± 1	0.02
Total days of ovarian stimulation	11 ± 1	11 ± 2	0.92
Total random start cycles	24 (100%)	16 (84.2%)	0.08
Antral follicle count	14 ± 10	15 ± 9	0.45
Total dose of gonadotropins (IU)^*^	4770 ± 1480	3846 ± 1438	0.04
Total days of antagonist use	6 ± 1	5 ± 1	0.2
Maximum serum estradiol (pg/mL)	1667 + 935	1981 + 1089	0.25
Oocytes retrieved	19 ± 14	22 ± 12	0.57
MII oocytes retrieved	12 ± 10	15 ± 8	0.2
Oocyte cryopreservation cycles	13 (54.2%)	18 (94.7%)	0.005
MII oocytes vitrified	15 ± 12	17 ± 9	0.51
Total oocytes vitrified	19 ± 15	21 ± 10	0.7
Embryo cryopreservation cycles	11	1	0.005
Fertilization rate (2PN/MII)	0.86 ± 0.14	0.5	—
2PN vitrified	1 ± 2	0	—
Blasts vitrified	3 ± 4	3	—

Values are expressed as mean ± standard deviation and percentages are represented in parentheses where appropriate. Statistical comparison was not performed for embryo cryopreservation cycles because only one was completed in 2019.

2PN, 2 pronuclei embryo stage; MII, metaphase II.

Notably, 11 cycles in 2020 (45.8%) started antagonist injections on days without monitoring, compared with 4 in 2019 (21.1%, *p* > 0.05). Nine cycles in 2020 (37.5%) utilized a “blind” oocyte maturation trigger without bloodwork and ultrasound monitoring on the day of trigger. No cycles used blind triggers in 2019. In 2020 and 2019, there was similar use of GnRH agonist triggers alone (2/24 [8.3%] vs. 5/19 [26.3%], *p* > 0.05) and hCG triggers alone (11/24 [45.8%] vs. 13/19 [68.4%], *p* > 0.05), but there were significantly more dual agonist/HCG triggers used in 2020 (11/24 [45.8%] vs. 1/19 [5.3%], *p* = 0.005).

Despite protocol modifications, there was no difference in the number of oocytes retrieved between 2020 and 2019 (19 ± 14 vs. 22 ± 12, *p* > 0.05). These findings persisted when results were adjusted for age, BMI, AMH, and antral follicle count (*p* = 0.68). In 2020, only three cycles retrieved less than five oocytes, and in 2019, two cycles retrieved less than five oocytes (*p* > 0.05). There was no difference in the number of mature oocytes retrieved (12 ± 10 vs. 15 ± 8, *p* > 0.05) or the number of mature oocytes vitrified (15 ± 12 vs. 17 ± 9, *p* > 0.05).

Among those who cryopreserved embryos at the blastocyst stage, there was no difference in the number of blastocysts cryopreserved in 2020 versus 2019 (3.6 ± 4.1 vs. 3), although only one embryo banking cycle was completed in the 2019 cohort. Three embryo cryopreservation cycles in 2020 froze at 2PN stage, one of which was planned for 2PN cryopreservation for future possible preimplantation testing, and the remaining eight cycles froze at the blastocyst stage.

The 2019 cycle embryo cryopreservation cycle froze at blastocyst stage. Five embryo banking cycles included PGT-A in 2020, with an average of 1.2 ± 0.8 euploid embryos frozen. PGT-A was not performed for the one embryo cryopreservation cycle in 2019. There were no cases of ovarian hyperstimulation in 2020 or 2019. One patient in 2019 was admitted after FP for management of tubo-ovarian abscess, and one patient in 2020 was evaluated in the emergency department for sickle cell-related pain crisis following FP.

## Discussion

In our small retrospective cohort study examining FP outcomes before and during the initial outbreak of the COVID-19 pandemic, we saw more FP cycles during the first wave of the COVID-19 pandemic than during the same period in 2019, in part reflecting absorption of patients from other centers unable to continue care. Continuing urgent FP throughout the pandemic required rapid modifications to our standard practices to care for these vulnerable patients. Despite modifications, there was no difference in cycle outcomes between 2019 and 2020.

To mitigate risks to patients and staff during COVID-19, we modified our protocols to minimize in-person visits, leading to increased telehealth consultations, significantly fewer monitoring visits, and administration of antagonist and triggers on unmonitored days, with one-third of patients triggered “blindly.” While our center decreased the number of in-person encounters to reduce the risk of viral transmission of SARS-CoV-2, outcomes for the 2020 cohort were optimal and comparable to historical controls.

Given these more limited visits in 2020, however, there were fewer opportunities for dose titration, which may have led to higher total gonadotropin doses during each cycle. Random start protocols, frequently implemented in our center and represented in these data, are standard practice in FP to minimize delays in urgent gonadotoxic therapy and are associated with higher total doses of gonadotropin use.^4,9–11^ Even with higher gonadotropin dosing, more intensive monitoring may not be indicated. Studies examining long-term follow-up of patients with breast cancer suggest that gonadotropin dosing does not affect disease-free survival, regardless of whether adjunctive medications are used for estradiol suppression.^9,12–15^

While evidence supporting current monitoring practices is limited,^16^ there is clear evidence that random start protocols are particularly well suited for streamlined monitoring and there is likely an important role for minimizing visits overall for medically compromised patients pursuing FP.

Patients undergoing urgent FP may be at a higher risk of complications from COVID-19 and other exposures given underlying malignancy or chronic conditions. Their underlying diagnosis of cancer or other acute condition requires numerous hospital visits, and limiting the number of trips to a fertility clinic will by definition increase the accessibility of this care. While our study represents a small number of patients, and larger numbers of prospective data are needed, our data add to the evidence that more limited monitoring in COH can be implemented without compromising outcomes.^17,18^

Although the COVID-19 pandemic may have further limited short-term access to FP through closures and delays in care, our findings highlight opportunities to make care more accessible long term. Telemedicine visits helped limit in-person visits, making care less burdensome for patients and widening the patient base able to access FP. By limiting monitoring with ultrasound and blood tests, our study's modifications may also represent a means to make FP more cost-effective for patients. More than 90% of patients identify cost as a major barrier to pursuing FP,^19^ and only 10 states currently mandate insurance coverage for medically indicated FP.^20^

Prospective examinations of cost and outcomes in reduced-monitoring settings are needed. However, to our knowledge, no studies have examined cost effectiveness of reduced monitoring in COH,^16^ and few studies have examined cost-effectiveness strategies in FP overall.^21^ The potential cost savings from minimizing in-person visits and laboratory expenditures may be impacted by the increased use of gonadotropins, as cost-effectiveness analyses of IVF protocols have suggested that these drugs are a major driver of cost.^22,23^ Future studies are needed to examine the cost effectiveness of minimized monitoring in COH.

Finally, significantly more patients pursued embryo cryopreservation rather than oocyte cryopreservation in 2020 compared with 2019 despite similar rates of partnership in both cohorts. The decision to cryopreserve embryos in lieu of oocytes may reflect a shift in reproductive decision making during the pandemic. A recent study by the Guttmacher Institute suggested that >40% of women have changed their reproductive life plans in response to the pandemic.^24^ Higher rates of embryo freezing in 2020 may reflect a COVID-driven shift in family building goals, increased confidence in relationships, or draw toward more established FP methods.

This study has several limitations, including its small sample size located at a single institution and retrospective design, limiting the generalizability of our findings. Importantly, our data are not the first to suggest that limited monitoring can lead to optimal outcomes. There is a need for further research to better elucidate the appropriate amount of monitoring and prospectively validate that decreased monitoring can lead to equivalent outcomes.

Given the lasting impacts of COVID-19 and the constant resurgences of new variants, many of our protocols have remained in place. The risk of SARS-CoV-2 infection for vulnerable populations is likely to persist in the near future, and clinics must be prepared to pivot and modify care for other urgent/emergent indications.^25^ Despite the limitations of our study, our findings provide a blueprint for how urgent FP care may be adapted long term without compromising outcomes.

The first wave of the COVID-19 pandemic led to massive disruptions to medical care worldwide, including an unprecedented ASRM-recommended suspension of routine fertility care. Importantly, despite local and national restructuring of care to preserve resources and protect the community, the ASRM encouraged centers to offer uninterrupted FP care for patients with cancer and other fertility-threatening conditions. This emphasis on the importance of FP despite the pandemic further highlights the essential and urgent nature of this care. Although planned cancer therapy has continued throughout the COVID-19 pandemic, cancer patients have been noted to be at increased risk of morbidity from SARS-CoV-2 infection.^8,26^ While our center was able to continue operations, others in our region could not.

The need to urgently absorb patients from neighboring centers highlights the importance of maintaining regional referral networks in the event of emergencies, to prevent interruptions in care. Given widespread closures of fertility clinics in tandem with preexisting barriers to care, COVID-19 has likely further limited access to FP care.^27,28^ Despite this ongoing pandemic and its widespread effects on medical care, FP must remain accessible and safe. Our small retrospective study confirms the feasibility of urgent FP during the outbreak of the pandemic, and our results suggest ways to minimize monitoring and streamline stimulation protocols in the future.

## Data Availability

The data generated and analyzed during the current study are included in the published article.

## Abbreviations Used

2PN: two pronuclei
AMH: anti-Mullerian hormone
ASCO: American Society of Clinical Oncology
ASRM: American Society for Reproductive Medicine
BMI: body mass index
COH: controlled ovarian hyperstimulation
E2: estradiol
FP: fertility preservation
FSH: follicle-stimulating hormone
hCG: human chorionic gonadotropin
PGT-A: preimplantation genetic testing for aneuploidy
PGT-M: preimplantation genetic testing for monogenic diseases
PPE: personal protective equipment
